# Case report of pelvic tuberculosis resulting in Asherman’s syndrome and infertility

**DOI:** 10.1186/s40738-019-0061-0

**Published:** 2019-08-01

**Authors:** Mary Louise Fowler, Shruthi Mahalingaiah

**Affiliations:** Department of Obstetrics and Gynecology, Boston Medical Center, 85 E Concord St 6th Floor, Boston, MA 02118 USA

**Keywords:** Infertility, Tuberculosis, Asherman’s syndrome, Intrauterine adhesions

## Abstract

Approximately one-third of the world’s population is infected with *Mycobacterium tuberculosis*, and it is a leading cause of infertility in endemic countries. The global incidence of tuberculosis (TB) is growing at approximately 0.4% per year, and much faster in sub-Saharan Africa. TB causing fertility is rare in developed countries. We present a case of genital tuberculosis causing Asherman’s syndrome and resultant infertility. The patient is a 34-year-old P0 who presented to care after a prolonged period of secondary amenorrhea and infertility. She underwent a hysterosalpingogram which demonstrated no free spill and a diagnostic hysteroscopy which had findings of mottled endometrium. Pathology returned positive for *Mycobacterium tuberculosis*. The patient was treated with 9 months of antituberculous therapy. While she has not yet succeeded in becoming pregnant, the patient has started to notice cyclic spotting, indicating possible return of menses. This case highlights the importance of TB treatment and considering TB in patients who present with unexplained infertility.

## Background

Tuberculosis (TB), most commonly from *Mycobacterium tuberculosis* bacteria, is one of the top ten causes of death worldwide, and, in 2017, was responsible for the deaths of 1.6 million people [1]. Additionally, there were over 10 million new cases of TB in 2017 alone. While TB is most commonly thought of as affecting the pulmonary system, TB can affect any part of the body. Urogenital TB is the third most common form of extrapulmonary TB [2]. Genital tract TB usually arises from hematogenous spread from a pulmonary or other non-genital source. Most often, TB of the female genital tract involves the fallopian tubes, the endometrial cavity and the ovaries. Importantly, genital tract TB has been associated with up to 21% of infertility cases in developing countries, due to tubal obstruction or adhesions in the uterine cavity [3, 4] and is even higher in patients with tubal factor infertility [5]. Symptoms of genital TB are often absent – most often, patients present with infertility, pelvic/abdominal pain or menstrual disturbances [6]. Asherman’s syndrome refers to intrauterine adhesions accompanied by symptoms of infertility or amenorrhea. The syndrome spans a spectrum of findings – from minimal disease with only thin strands of tissue stretched across the uterine cavity while severe disease is characterized by complete obliteration of the cavity with densely adherent uterine walls. Genital TB is commonly associated with severe intrauterine adhesions, with complete obliteration of the uterine cavity [6, 7].

## Case presentation

**Fig. 1:**
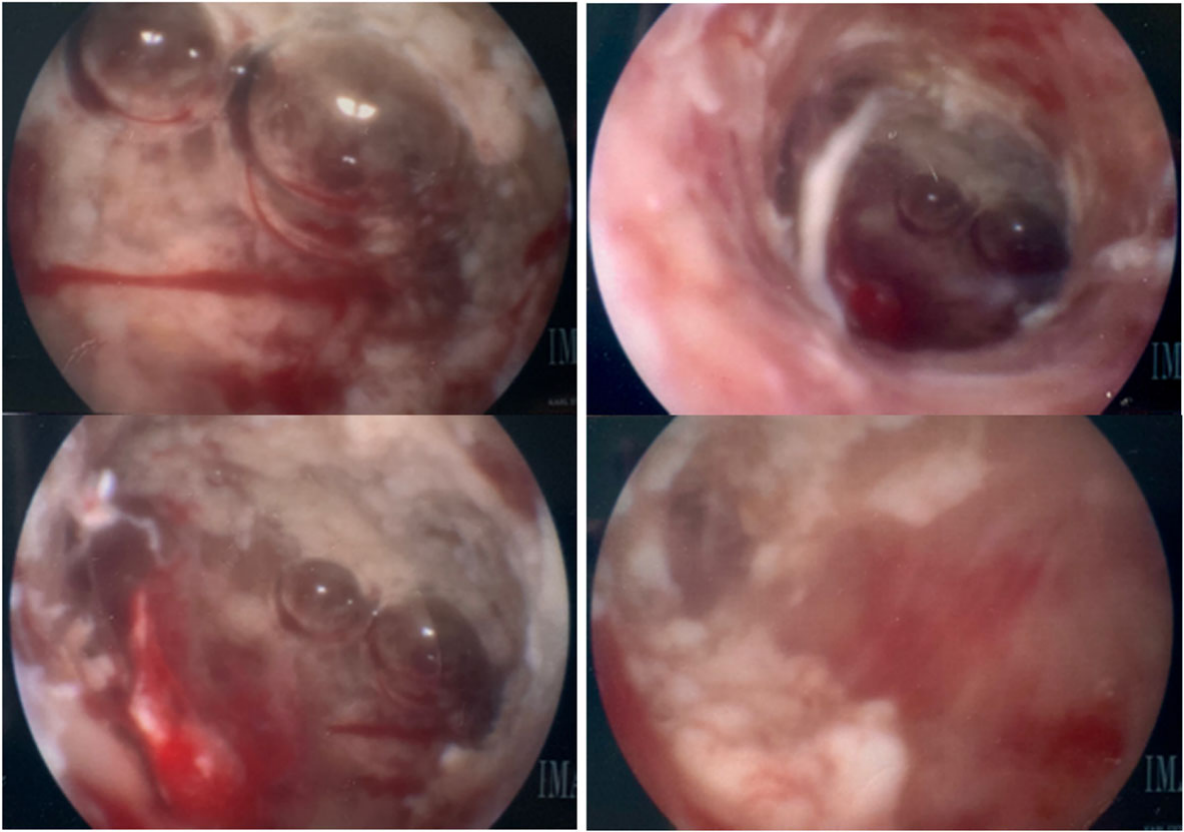
Images of uterine cavity captured from diagnostic hysteroscopy, November 2017

This patient is a 34-year-old non-obese Ethiopian P0 who presented for care after prolonged (5–6 years) secondary amenorrhea and infertility. She had no past medical history. Past surgical history significant for a myomectomy with Pfannenstiel incision in 2009 in Ethiopia for subserosal fibroids. She immigrated to the United States in 2010. In terms of family history, the patient’s older sister had positive TB 20 years ago, for which her sister was treated. No one else in the family was treated for TB. A work up was completed for secondary amenorrhea. At this time there was no evidence of adhesions. A hormonal evaluation was obtained and she was worked up for amenorrhea as well as routine infertility labs, including TSH, prolactin, FSH, LH, AMH and estradiol to assess the entire axis. There was a low ovarian reserve based on AMH (0.61), however the normal values of FSH, LH, estradiol and progesterone levels suggested recent ovulation therefore leaning to more of a uterine pathology. On ultrasonography, the uterus measured 7.5 × 3.5 × 6.2 cm with an unremarkable myometrium. The endometrial stripe measured 4 mm and was noted to be uniform. A hysterosalpingogram performed at age 29 demonstrated no opacification of the left fallopian tube and no intraperitoneal free spill from the right fallopian tube, suggesting occlusion. In November of 2017, the patient underwent a diagnostic hysteroscopy, with findings of mottled endometrium particularly near the right ostia across the fundus. (See Fig. 1). Endometrial curretings from this procedure were collected and sent for TB testing. The pathology from this demonstrated necrotizing granulomas with acid-fast bacilli positive and culture positive for *Mycobacterium tuberculosis*. A chest x-ray was performed but was negative for any radiographic evidence of active pulmonary TB.

At this time, the patient was started on 2-month course of 4-drug Rifampin, Isoniazid, Pyrazinamide, and Ethambutol (RIPE) therapy followed by 4 months of rifampin and isoniazid. After 6 total months of treatment, the patient returned for a repeat endometrial biopsy. Results from this biopsy demonstrated positive acid-fast bacilli on Kinyoun stain but the cultures did not grow. Per an Infectious Disease consult, the patient was continued on rifampin and isoniazid for an additional 3 months. A second endometrial biopsy was performed in September 2018, which returned culture negative and acid-fast bacilli negative. The patient is currently undergoing treatment an estrogen-priming protocol to attempt to regrow endometrium, and at this point she has begun to have slow return of cyclic spotting after 2 cycles. It remains to be seen if this patient will have potential for future fertility.

## Discussion

Genital TB is an important cause of female infertility, particularly in developing countries, but is not routinely encountered in the United States. Current data demonstrates fallopian tube involvement in 90–100% of cases, most commonly bilaterally, followed by endometrial involvement in 50–70% of cases. With fallopian tube involvement, one is most likely to find congestion, hydrosalpinx or tubo-ovarian masses. Presence of endometrial tuberculosis signifies infection of the fallopian tubes. In a study done by Sharma et al. [6], 28 women underwent hysteroscopy with or without laparoscopy for suspected Asherman’s syndrome. Of these women, 67.8% had a past history of TB and all women had either primary (67.8%) or secondary (32%) infertility. Of all of these women, various grades of adhesions were discovered and only 4 had open ostia.

The diagnosis should be suspected in patients with clinical manifestations such as infertility, pelvic or abdominal pain, as well as pertinent history such as history of prior TB infection or known/possible exposure to TB. In terms of imaging, a hysterosalpingogram (HSG) may show obstruction of the tubes or uterine cavity constriction, representing adhesions. Other characteristic findings on HSG in women with genital TB: beading, sacculation, sinus formation and pipe-stem patterning [8]. Endometrial biopsy can be collected for histology and culture as well as staining for acid-fast bacilli and mycobacterial culture. Interestingly, menstrual fluid is more sensitive than biopsy specimens for the diagnosis of TB endometritis [9]. However, it should be noted that this mode of culturing may not be possible when amenorrhea has resulted from the infection. Patients with genital TB should be treated with antituberculous therapy consisting of RIPE (Rifampin, Isoniazid, Pyrazinamide, Ethambutol) for two months followed by rifampin and isoniazid for 4 months [10]. Surgery may be necessary with total abdominal hysterectomy and bilateral salpingo-oophorectomy being the definitive approach. Despite treatment of pelvic TB, the associated infertility is often irreversible. In a study done by Mondal et al. [4], 56 patients with confirmed genital tuberculosis were studied with a mean age of 25.6 years. After treatment, 9 patients conceived of which 8 suffered spontaneous abortions and only one patient had a successful pregnancy.

Unfortunately, most women with genital TB are not only presenting with infertility but also have poor prognosis for fertility despite assisted reproductive therapies (ART). The conception rate is notably low at 19.2% [11]. Parikh et al. found in-vitro fertilization with embryo transfer to be the only possibility for some women whose endometrium was not damaged, with a pregnancy rate of only 16.6% per transfer [12]. However, a study by Jindall et al. [13] found that for patients with genital TB, once treated with anti-TB treatment and ART, had an overall pregnancy rate of 60%.

It is clear that the most important first steps for a patient presenting with genital TB and infertility are anti-TB treatment and likely ART. Early diagnosis and treatment is crucial for these patients to attempt to correct the often irreversible structural scarring.

## Data Availability

Not applicable.

## Abbreviations

AMH: Anti-Mullerian Hormone
ART: Assisted Reproductive Therapies
FSH: Follicle Stimulating Hormone
HSG: Hysterosalpingogram
LH: Luteinizing Hormone
RIPE: Rifampin, Isoniazid, Pyrazinamide, Ethambutol
TB: Tuberculosis
TSH: Thyroid Stimulating Hormone
